# Defining safety net hospitals in the health services research literature: a systematic review and critical appraisal

**DOI:** 10.1186/s12913-021-06292-9

**Published:** 2021-03-25

**Authors:** Jennifer L. Hefner, Tory Harper Hogan, William Opoku-Agyeman, Nir Menachemi

**Affiliations:** 1 Division of Health Services Management and Policy, School of Public Health, The Ohio State University, Columbus, OH USA; 2 School of Health and Applied Human Sciences, University of North Carolina at Wilmington, Wilmington, NC USA; 3 Department of Health Policy and Management, Indiana University Richard M. Fairbanks School of Public Health, Indianapolis, IN USA

**Keywords:** Hospitals, Safety net providers, Disparities, Systematic review

## Abstract

**Background:**

The aim of this study was to identify the range of ways that safety net hospitals (SNHs) have been empirically operationalized in the literature and determine the extent to which patterns could be identified in the use of empirical definitions of SNHs.

**Methods:**

We conducted a PRISMA guided systematic review of studies published between 2009 and 2018 and analyzed 22 articles that met the inclusion criteria of hospital-level analyses with a clear SNH definition.

**Results:**

Eleven unique SNH definitions were identified, and there were no obvious patterns in the use of a definition category (Medicaid caseload, DSH payment status, uncompensated care, facility characteristics, patient care mix) by the journal type where the article appeared, dataset used, or the year of publication.

**Conclusions:**

Overall, there is broad variability in the conceptualization of, and variables used to define, SNHs. Our work advances the field toward the development of standards in measuring, operationalizing, and conceptualizing SNHs across research and policy questions.

**Supplementary Information:**

The online version contains supplementary material available at 10.1186/s12913-021-06292-9.

## Background

Safety net hospitals (SNHs), often located in poor and underserved communities, tend to serve large populations of racial and ethnic minorities and face unique challenges in providing high quality care in resource constrained environments [1, 2]. State and local municipal budget shortfalls and cuts to Medicaid reimbursements increase pressure on SNHs, put them at greater risk of closing or privatizing [3], and threaten their ability to meet their communities’ needs [4]. As a result, there have been calls for an expanded program of research to identify ways to assure the viability and increased effectiveness of SNHs [3]. However, such a research program must first overcome the lack of consensus among researchers on how SNHs are defined and empirically categorized [5, 6].

Anecdotally, researchers have operationalized SNHs in their studies in a variety of ways and frequently justified their chosen approach by stating that there is a lack of consensus on how to empirically identify such hospitals [7–11]. Two studies, published a decade apart, selected three frequently used definitions of SNHs and found differences in the sample of hospitals across definitions, both in terms of organizational characteristics and facility financial viability [5, 6]. Thus, it has been suggested that a SNH definition should be chosen based on the empirical or conceptual aims of a given study [6]. Problematically, this could lead to inter-study variability with respect to hospitals that are considered “safety-net” resulting in ambiguity about the generalizability of findings and the potential policy solutions that can address the needs of vulnerable groups.

Working towards a consensus around how to identify SNHs, the purpose of this study is to (1) identify the range of ways that SNHs have been defined by identifying the empirical operationalizations used in the literature, (2) explore whether patterns exist in the variability in SNH definitions, and (3) determine the extent to which studies focused on similar topics (e.g., financial performance, or quality of care) use comparable empirical definitions of SNH. These aims are accomplished via a PRISMA-guided [12] systematic review of SNH articles published in the health services literature between 2009 and 2018. Categorizing SNH definitions advances the field of health services research (HSR) toward the development of standards in measuring, operationalizing, and conceptualizing SNHs across research and policy questions.

Despite commentaries and some evidence that SNH definitions produce variability in samples of SNH, researchers continue to deviate in how SNH are defined and there is no published work aimed at moving toward a consensus. Identifying the landscape of SNH definitions is of importance because of myriad policies aimed at relieving the financial burden faced by hospitals that serve America’s poorest and sickest communities. To understand the impacts of these policies on SNHs, a standard way to identify these facilities is needed. While this study focuses on the published research literature, SNH are also defined differently across state and federal government programs that provide incentive payments to SNHs [13–15]. To inform policy and practice, we have conducted the first systematic review summarizing the categorization and operationalization of definitions that identify hospitals that make up the U.S. healthcare safety net. This study advances the literature on SNHs toward a path of improved measurement precision and comparability among policy and health service studies in the U.S.

## Methods

Our research methodology included all required elements of the Preferred Reporting Items for Systematic Reviews and Meta-Analyses (PRISMA) checklist for systematic reviews except for an assessment of the quality of the evidence because the focus of our review was on the variety in operationalizations of SNHs not a summary of the findings across studies [12]. Therefore, assessing the risk of bias within and across studies is not applicable to our study aims. Through the PRISMA review process we identified common definitions and then categorized the definitions based on the types of hospital characteristics considered in the definition.

### Search strategy

We initially searched ProQuest for articles published between 2009 to 2018, supplemented by ABI INFORM, Business Source Complete (EBSCO host), PubMed, and Google Scholar. We restricted the search to articles published in the decade following the passage of the Affordable Care Act and subsequent federal payment reforms given that these changes heightened interest in the performance of SNHs. All articles that focused on hospitals and safety-net providers were retrieved and indexed at the time of the literature search (November 2018). The keywords used in the search criterion included: “safety net”, “safety-net”, “safety-net hospital” and “safety net hospitals”. This keyword search was applied to article abstracts. These sets of citations were then crossed-checked with “hospitals” as the subject area. Given the known limitations of keyword database searches, we further performed a hand search of the reference lists of articles that were identified in the electronic search and met our inclusion criteria, which are outlined in the next section. The full electronic search strategy for ProQuest was*: Keywords = safety net OR safety-net OR safety-net hospitals OR safety net hospitals in Abstract, with filter selections of Source-Scholarly Journals, Full text/Peer reviewed, Publication date 01-01-2009 to 10-29-2018, Document type-Article, Subject-Hospitals, Location-United States-US including individual states and cities, Language-English*.

### Study selection and eligibility criteria

All abstracts derived from these searches were screened and analyzed based on the inclusion criteria: (1) peer-reviewed article, (2) in the English language, (3) with a focus on safety net hospitals (excluding other safety net healthcare settings), and (4) relevant to the field of health services research (HSR). We follow AcademyHealth, the HSR professional organization in defining HSR as a “multidisciplinary field of scientific investigation that studies how social factors, financing systems, organizational structures and processes, health technologies, and personal behaviors affect access to health care, the quality and cost of health care, and ultimately our health and well-being” [16]. Full texts of all articles included at this step were independently reviewed by study authors and discussed at team meetings. Articles were excluded at this step if the study did not empirically measure SNHs or if the level of analysis was not the hospital, i.e. patient-level data analysis. Analyses of patients who use SNHs were not applicable to our hospital-level study aim of identifying how articles operationalize SNHs when conducting hospital-level analyses.

### Data extraction

Two authors (JLH and THH) independently reviewed each article and extracted relevant data from the methods sections of each published manuscript. Consensus was used to mediate any disagreement in coding. The main variable extracted from each article was the operational definition of SNHs and the lines of text that described the process of choosing a definition, if applicable. Additional extracted data included the dataset used in the analyses, the key variables in the study (both independent and dependent), the journal name (categorized into health services journals, policy journals or clinical journals) and the publication year. Further, consistent with the definition of HSR described above, we categorized the key outcome variable of each included study as related to “costs,” “quality,” or “access.”

### Data synthesis

The process of data synthesis included the identification of common definitions across articles and the flagging of similar but not identical definitions, for example the percent uninsured versus the proportion of charges for charity care. A five-category classification system adapted from McHugh et al. [5] was employed to group common operational definitions thematically based upon whether the definition focused on: facility characteristics (e.g., status as a public or teaching hospital), patient case-mix (e.g., socioeconomic and/or health status); Medicaid Disproportionate Share Hospital Payment (DSH) status, Medicaid caseload (e.g., percent of inpatient discharges that are Medicaid), and/or level of uncompensated care (e.g., proportions of uninsured patients, self-pay patients, or charity care). Each of the operational definitions was attributed to one of these thematic categories or a combination of two or more categories.

### Data analysis

As part of our analysis, we examined whether an association existed between each of the thematic categories and the category of outcome variables from each included study. To do so, we examined the degree of concentration of articles within each thematic category to each outcome domain by calculating a modified Herfindahl-Hirschman Index score. Doing so allowed us to quantify the extent to which any definition was most consistently used within studies that examined cost, quality, or access. We categorized concentration consistent with established conventions whereby a given definition was considered highly concentrated with a score above 0.25 and moderately concentrated between 0.15 and 0.25 [17]. To document the selection of SNH operational definitions across studies, we created three binary variables coded as 1 if each were present in the article when the authors presented their SNH definition: a reference was cited, the definition choice was justified with supporting text, and authors noted the variety of definitions in the literature.

## Results

**Fig. 1:**
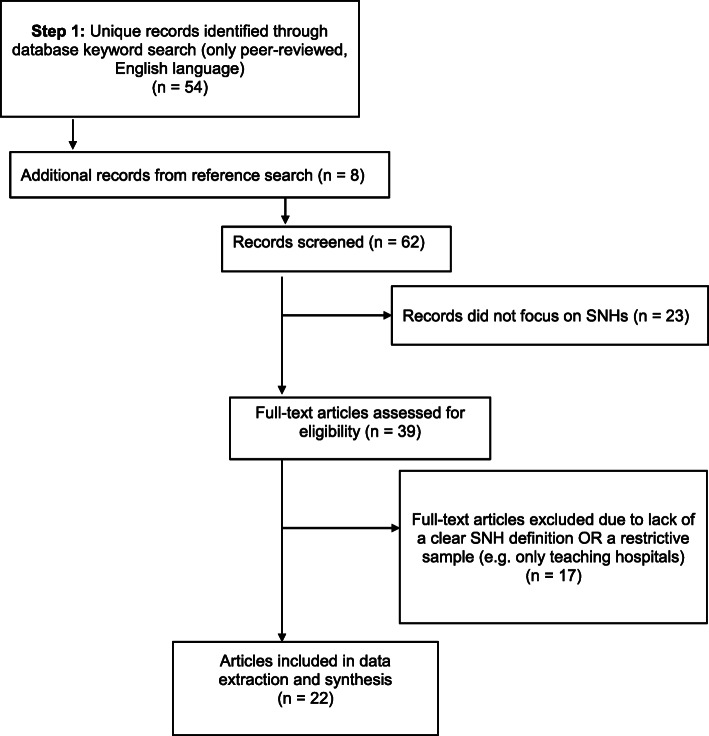
PRISMA Flow Diagram

Our database search yielded 54 articles and 8 additional articles were identified using the hand reference search (see Fig. 1). A total of 23 articles were excluded when reviewing title and abstracts because they did not focus on SNHs. Of the 39 full-text articles reviewed, 17 were excluded due to a lack of a clear SNH definition or a restrictive sample, e.g. only teaching hospitals. Overall, the search process resulted in 22 articles that met inclusion criteria. A list of the 22 articles and data extraction variables is provided in the Appendix.

In Table 1, we present article characteristics of the 22 articles. Almost half of the articles were published in health services journals (45%), about a third were published in policy-related journals (32%) and a quarter of studies were published in clinical-related journals (23%). The most frequent data source used among included studies to identify SNHs was the American Hospital Association (AHA) annual survey of U.S. hospitals, used by 59% of the articles. The Medicare Cost Report was used 27% of the time, followed by the Healthcare Cost and Utilization Project (HCUP) (25%). Six data sources were used only once. Among the five thematic categories, Medicaid caseload was used in the operational definition of a SNH for 13 of the 22 articles (59%). DSH payments was used in 8 articles, and uncompensated care, facility characteristics, and patient case mix were part of the SNH definition in 7 (32%), 5 (22%), and 2 (9%) articles respectively.

**Table 1:** Description of included articles (*n* = 22)

Variables	Frequency (%)
**Publication Year**	
2009–2012	6 (27%)
2013–2015	7 (32%)
2016–2018	9 (41%)
**Journal Type**	
Health Services journals	10 (45%)
Policy journals	7 (32%)
Clinical journals	5 (23%)
**Data Source**^a^	
American Hospital Association (AHA)	13 (59%)
Medicare Cost Reports	6 (27%)
Health Care Utilization Project (HCUP) and other inpatient discharge databases	5 (23%)
National Inpatient Sample (NIS)	2 (9%)
Virginia Health Information discharge data	1 (5%)
CA Office of Statewide health planning and development (OSHPD)	1 (5%)
Agency for Healthcare Research Socioeconomic Status Index	1 (5%)
Centers for Medicare and Medicaid Services Impact File	1 (5%)
**Five category classification system**^a^	
Medicaid Caseload	13 (59%)
Disproportionate Share (DSH) payments	8 (36%)
Uncompensated care	7 (32%)
Facility characteristics	5 (23%)
Patient case mix	2 (9%)
**Outcome of focus among included studies**^a^	
Quality	17 (77%)
Cost	8 (36%)
Access	2 (9%)
**Inclusion of a source or rationale for SNH definition**	
Accompanying reference was cited	17 (77%)
Definition choice was justified with supporting text	12 (55%)
Authors noted the variety of SNH definitions in the literature.	11 (50%)

^a^Some articles included more than one definition of SNH, more than one dataset, and/or focused on more than one outcome. Thus, sums do not add up to 100%

The most common outcome studied among included articles was categorized as quality and pertained to 17 articles (77%); 8 articles focused on cost (36%) and 2 on studies focused on access (9%). Seventeen of the 22 articles (77%) referenced others’ published work to accompany selection of a SNH definition, and 12 of these 17 went a step further to supply supporting text to justify the selection of their definition from among other options. Additionally, in 11 of the 22 articles the authors noted the variety of SNH definitions in the literature, and each of these 11 also included a source or rationale for selection of a particular definition.

In total we identified 11 unique operational definitions of SNHs across the 22 articles (Table 2). The single most commonly used operational definition was the top quartile (or top 20%) of DSH payments, and it appeared 7 times among included articles. Definitions based on Medicaid caseloads were collectively very common and included the following specific definitions: Medicaid and Uninsured Inpatient Discharges operationalized as hospitals in the top quartile (appeared 5 times among included articles), and Medicaid Inpatient discharges operationalized as one standard deviation above the state median or mean (appeared 4 times). In Table 2, we also present the distribution of operational definitions across the five thematic categories. Among these categories Medicaid caseload was the most frequently used thematic category followed by uncompensated care and facility characteristics. Lastly, 3 of the 11 unique operational definitions of SNHs used a combination of two or more categories. A measure of Medicaid caseload was used in all three combination definitions.

**Table 2:** Frequency of use of each operational definition of Safety Net Hospital (SNH) and corresponding thematic categories among included articles published 2009–2018

	Operational Definition of SNH	Frequency of use	Thematic Categories^a^				
			Medicaid caseload	Uncompensated care	Facility characteristics	DSH payments	Patient case mix
1	DSH payment index (top quartile/top 20%).	7				X	
2	Medicaid and Uninsured Inpatient Discharges (top quartile)	5	X	X			
3	Medicaid Inpatient Discharges (one standard deviation above the state median/mean).	4	X				
4	Medicaid Inpatient Discharges (one standard deviation above state mean) OR Non-Federal Government Hospitals (Public Hospitals).	3	X		X		
5	AHRQ SES Index.	1					X
6	The ratio of bad debt plus charity care (top decile) OR uncompensated care expenses	1		X			
7	Teaching COTH Member OR Non-Federal Government (Public Hospitals).	1			X		
8	Medicaid Inpatient Discharges (top quartile) OR DSH status (y/n) OR proportion of charges for charity care.	1	X	X		X	
9	Percentage of patients eligible for Supplemental Security Income (SSI)	1					X
10	Non-Federal Government Hospitals (Public Hospitals)	1			X		
11	Medicare uncompensated care burden (top quartile)	1		X			
	**Total**	26	4	4	3	2	2

^a^adapted from McHugh, 2009

In Table 3, we present data that explores potential patterns in the use of definition categories across the 22 articles. We found no obvious patterns between the definitional categories and select article characteristics including datasets used, the journal type, or the year of publication. Specifically, articles that identified SNHs using the definition category of ‘Medicaid caseload + uncompensated care’ did not utilize a common dataset – in fact this definition was operationalized using 4 different datasets. Additionally, studies using this definition category were published in either health services or clinical-related journals across all study years. Similarly, we observed no pattern in dataset, journal type or year for all definition categories that appeared more than once in our sample.

**Table 3:** Frequency of SNH definition(s) used by article characteristics (*n* = 22 articles)

	Data Sources					Journal Type			Year Categories		
	Cost Reports	AHA	HCUP	NIS	Other dataset	Policy- related	Health services	Clinical-related	2009–2012	2013–2015	2016–2018
Medicaid caseload		3			1	2	2		3	1	
Uncompensated care		1					1		1		
Facility characteristics		1					1		1		
DSH payments	4	3			1	4	2	2		4	4
Patient case mix	1				1	1		1			2
Medicaid caseload + facility characteristics		3	1			1	1	1	2		1
Medicaid caseload + uncompensated care		1	2	2	2		3	2		2	3
Uncompensated care + DSH payments + Medicaid caseload					1		1		1		

SNH is Safety Net Hospital, AHA is American Hospital Association Annual Survey of Hospitals, HCUP is Health Care Utilization Project, NIS is National Inpatient Sample

In Table 4, we present the concentration of each SNH definition category within the main outcome variable used among the 22 included articles. Among the studies that examined cost outcomes there was moderate concentration in definition categories, with ‘DSH payments’ and ‘Medicaid caseload + uncompensated care’ used most commonly. Among the studies whose main outcome variable was categorized as “quality,” there was very low concentration of any single thematic category used (e.g., there was much variability of definitions used among studies focused on quality). Lastly, among studies focused on access, there was moderate concentration of definitions relying on the thematic category of Medicaid caseload; but notably there were only two studies focused on access.

**Table 4:** Concentration of thematic categories of SNH definitions among studies with similar outcomes measures (*N* = 22 articles)

Thematic Categories:	Main Outcome among included Studies					
	Cost	Concentration	Quality	Concentration	Access	Concentration
Medicaid caseload	0	0	4	0.06	0	0
Uncompensated care	0	0	1	0	0	0
Facility characteristics	1	0.02	1	0	0	0
DSH payments	4	0.25	3	0.03	0	0
Patient case mix	0	0	2	0.01	0	0
Medicaid caseload + facility characteristics	0	0	2	0.01	1	0.25
Medicaid caseload + uncompensated care	3	0.14	3	0.03	1	0.25
Uncompensated care + DSH payments + Medicaid caseload	0	0	1	0	0	0

## Discussion

Given the importance of SNHs to the health care safety net in the U.S., many have called for an expanded program of research to identify ways to assure the viability and increased effectiveness of SNHs. Such a research program must first overcome the lack of consensus among researchers on how SNHs are defined and empirically categorized. The objectives of this review were to identify the range of ways that SNHs have been empirically operationalized in the literature, identify any patterns across studies, and determine the extent to which studies focused on similar topics use comparable empirical definitions of SNHs.

Among the 22 studies identified in this systematic review, there were 11 unique SNH definitions including many similar, but not identical, operationalizations. Using level of DSH payments to identify SNHs was the most common thematic definition category, however, it was used in less than a third of included articles. There was no dominating definition category. This finding is consistent with the claims of authors in this field, as we found that in half the methods sections in this review the authors noted that there is no consensus on how to empirically identify SNHs. This lack of a standard SNH definition has implications for policy development and evaluation. For example, without agreement on how to define a SNH, a study that aims to assess the impact of value-based payments on clinical outcomes among SNHs cannot determine if findings reflect the true impact of the policy or are a function of the SNH definition used. This creates a situation in which policy could be crafted based on inconsistent information and researchers and policymakers do not obtain a clear evaluation of the effects of such policy.

Across all definition categories that appeared more than once in our sample, we observed no pattern in either the dataset used, journal type, or year of publication. This suggests that a given author’s decision to define SNH in a specific way is not based on consensus but rather on some unmeasured influence. Additionally, 23% of the studies in our review did not cite a reference to justify the choice of a SNH definition. This lack of standardization has resulted in a problem of measurement reliability between studies. In fact, previous research comparing three definitions has indicated that there are differences in the population of SNH’s identified using different SNH definitions [5, 6]. Stakeholders have an imperative to develop a richer understanding of how definitions used to measure SNH affect the conclusions of research and evaluation.

Another important finding to consider is the level to which certain definition categories are concentrated in studies measuring similar outcomes. Among the studies whose main outcome variable was categorized as related to “quality,” there was very low concentration of any single thematic definition category used. In the studies that examined “cost” outcomes there was moderate concentration in thematic definition categories, with ‘DSH payments’ and ‘Medicaid caseload + uncompensated care’ used most commonly. However, even though “cost” papers used ‘DSH payments’ most commonly, this definition category accounted for only four of the eights cost studies. In a 2013 “cost” study Joynt &Jha used DSH payments to define SNHs, identifying a sample of 769 U.S. hospitals [18]. A 2018 “cost” study by Bazzoli and colleagues used both facility characteristics and DSH status as SNH definitions – and identified 640 and 1194 hospitals respectively [19]. These findings support the argument above that a lack of consistency in SNH definition impacts the sample identified and calls in to question the generalizability of study findings and related policy implications.

### Limitations

When interpreting the results of our systematic review, some limitations should be noted. First, the search results are limited to the efficacy of the search terms and the databases utilized. Despite our use of bibliographic hand search to supplement our database search, we recognize the possibility that some potentially relevant articles were inadvertently omitted. How such omissions have affected our conclusions are not fully known but are unlikely to dilute the broad variability in definitions used by the published articles that we identified. A second limitation is that our ability to identify each SNH definition was a function of the details provided in each study’s methods section. It was challenging to categorize and compare SNH definitions for studies that did not clearly operationalize their SNH definition. When data extraction of the SNH definition was not clear, the study was flagged and the authors reviewed the exact wording within the article and determined if a consensus could be reached. If a consensus could not be met, we reached out to the corresponding author of the published study to ask for clarification regarding how a variable was operationalized (this occurred on one occasion).

### Future research

Our study highlights many outstanding research questions and opportunities for future inquiry. For example, is being a SNH a fixed hospital characteristic? If so, researchers should not be selecting different definitions based on their research question, as has been suggested in the literature [6]. Further, it is an open question as to whether it is sufficient to use a binary yes/no variable to identify SNHs in which each hospital identified as a SNH is lumped into one group. SNHs are a diverse group of institutions with varying organizational and financial characteristics [20]. Prior research has established that SNHs with a high DSH payment rate are likely to be larger, urban teaching hospitals that are part of a system, while SNHs with high uncompensated care costs are likely to be located in rural areas with a smaller service mix and lower profit margins [6]. Since SNHs are a heterogeneous group that varies based on definition, more research is needed to identify the potential types of SNHs beyond the standard dichotomy of SNH yes/no. Specifically, qualitative research should explore whether SNH administers consider their status as a safety net provider a fixed trait. And if so, how do they understand the current policy environment in which a hospital moves in and out of SNH status based on the definition used. Quantitative projects should include comparing the sample of SNHs identified by each of the definitions and developing a taxonomy of SNHs similar to other taxonomies in health services research.

## Conclusions

Overall, we find that there is broad variability in the types of variables used to define SNHs. In order to advance the literature toward improved measurement precision and comparability among policy and health service studies, more work is needed. SNH definition standardization is necessary because of myriad policies aimed at relieving the financial burden faced by hospitals that serve America’s poorest and sickest communities. Until consensus is reached, health services researchers should be more cautious about their choice of SNH definition and test how their chosen operationalization affects the sample of SNHs identified. At a minimum, researchers should conduct robustness checks of their findings across SNH definitions to minimize the negative implications of the lack of a standard definition.

## Supplementary Information

**Additional file 1.**

**Additional file 2.**

## Data Availability

Appendix 2 is an excel table of the most pertinent data extracted from the review articles.

## Abbreviations

SNHs: Safety net hospitals
PRISMA: Preferred Reporting Items for Systematic Reviews and Meta-Analyses
HSR: Health services research
DSH: Medicaid Disproportionate Share Hospital Payment
AHA: American Hospital Association
HCUP: Healthcare Cost and Utilization Project
